# A Design Architecture for Decentralized and Provenance-Assisted eHealth Systems for Enhanced Personalized Medicine †

**DOI:** 10.3390/jpm15070325

**Published:** 2025-07-19

**Authors:** Wagno Leão Sergio, Victor Ströele, Regina Braga

**Affiliations:** Computer Science Department, Federal University of Juiz de Fora, Juiz de Fora 36036-900, Brazil; regina.braga@ufjf.br

**Keywords:** eHealth, EHR, PHR, decentralization, provenance, simulation, API, personalized medicine

## Abstract

Background/Objectives: Electronic medical record systems play a crucial role in the operation of modern healthcare institutions, enabling the foundational data necessary for advancements in personalized medicine. Despite their importance, the software supporting these systems frequently experiences data availability and integrity issues, particularly concerning patients’ personal information. This study aims to present a decentralized architecture that integrates both clinical and personal patient data, with a provenance mechanism to enable data tracing and auditing, ultimately supporting more precise and personalized healthcare decisions. Methods: A system implementation based on the solution was developed, and a feasibility study was conducted with synthetic medical records data. Results: The system was able to correctly receive data of 190 instances of the entities designed, which included different types of medical records, and generate 573 provenance entries that captured in detail the context of the associated medical information. Conclusions: For the first cycle of the research, the system developed served to validate the main features of the solution, and through that, it was possible to infer the feasibility of a decentralized EHR and PHR health system with formal provenance data tracking. Such a system lays a robust foundation for secure and reliable data management, which is essential for the effective implementation and future development of personalized medicine initiatives.

## 1. Introduction

As information technologies continue to evolve, new tools are being created to support activities in various segments of society. In the healthcare sector, there is a growing transition from manually documenting patient health data to the use of digital medical record platforms. This shift is particularly critical as healthcare moves towards a more patient-centric model, where personalized medicine plays an increasingly vital role.

According to [1], a medical record can be defined as a single document that compiles a set of information, signals, and recorded images, based on facts, events, and situations related to the patient’s health and the care received. It has a legal, confidential, and scientific nature, facilitating communication between members of the multidisciplinary team and ensuring the continuity of care provided. In addition, medical records are essential for health management, including teaching and research, implementing and evaluating public policies, and documenting legal issues. Users of medical records include professionals directly involved in care and management, researchers, teachers, students involved in healthcare, and the patients themselves.

The medical record is a thorough collection of data concerning a patient’s health status and care journey [1]. It holds scientific, legal, and private significance, facilitating interaction among diverse professional teams and promoting ongoing care and support. Moreover, considering new practices related to preventive medicine, also known as medicine 2.0, these records are essential for monitoring patient health and serve as the dataset for tailoring medical interventions to individual patient needs, which is a tenet of personalized medicine.

Supporting this new approach to preventive medicine, we have several data points from sensors that track patient information. Consequently, the growing volume of health-related information generated by these emerging technologies—such as wearable devices and digital medical platforms—has made traditional manual practices in healthcare increasingly inadequate. Dependence on these outdated approaches can lead to higher expenses and decreased service quality due to challenges in maintaining accurate records. Technological tools are crucial for supporting medical professionals, enhancing data exchange, supporting preventive actions through data processing, and unifying information to boost the overall effectiveness of healthcare delivery [2].

Nevertheless, in recent years, numerous security incidents have impacted digital systems handling health information, raising concerns about both the accuracy of stored records and the risk of exposing patients’ private data [3].

In March 2020, the University Hospital of Brno in the Czech Republic was targeted by a cyberattack, likely ransomware, which forced it to shut down its IT network and severely compromised its ability to manage the COVID-19 outbreak and conduct testing [4]. Another incident involved Babylon Health in London, where a data breach in the GP at Hand app allowed a patient to access another patient’s appointment recordings due to a bug. Experts emphasize that even minor breaches can lead to future attacks, underscoring the importance of security audits [5].

The World Health Organization (WHO) declared in 2024 that it was in the process of collaborating with UN agencies to actively support Member States with technical assistance, norms, standards, and guidance to enhance the resilience of health infrastructure against cybercrime, including ransomware [6]. In addition, during the pandemic, the U.S. National Academic of Public Administration’s president stated that the coronavirus exposed the “challenging state of government IT systems” and underscored the need for federal investment in modern, integrated IT infrastructure [7]. It was said that institutionally, the current approach viewed IT as an overhead cost, always seeking to minimize it, rather than recognizing it as a fundamental tool in the 21st century that would deliver increased accountability, better outcomes, and improved citizen satisfaction. These declarations highlight recent concerns from official institutions about the urgent need to address major problems in the health IT domain.

However, several challenges related to medical records systems arise from their structural design and configuration. Typically, these platforms rely on a client–server model, where a central unit manages the majority of storage and processing tasks while users interact with the system through client interfaces. Although this setup is widely used and relatively straightforward, it has limitations. Additionally, these systems often lack robust mechanisms for tracking data access, making it challenging to identify and resolve issues or determine their root causes.

First, this type of architectural style has a serious security vulnerability due to its centralized operation. Applications based on cloud computing present problems such as high usage costs, a lack of autonomy in controlling the use of available data, and the need for an almost constant broadband connection [8]. Furthermore, centralized paradigms are vulnerable to massive information leaks if an attacker gains access to the system [9]. Finally, it is worth noting that many of these systems, when they experience data integrity problems, are not efficient in providing support for auditing the failures that have occurred [9], due to the lack of traceability control.

Therefore, architectures based on centrality have security vulnerabilities. These paradigms risk massive information leaks if breached, and they often lack effective data integrity and auditing support due to poor traceability control [9]. The majority of electronic systems for managing medical records are designed primarily for the use of healthcare institutions and professionals, often excluding functionalities that allow patients to contribute their own personal details and use these data to preventive actions considering his/her health. One of the main artifacts participating in this process is Electronic Health Records (EHRs). They serve as digital repositories designed to store an individual’s health information throughout their lifetime [10]. Their main purpose is to preserve uniform medical history data, enabling seamless information exchange among different healthcare entities through a shared health data architecture and supporting preventive medicine.

On the other hand, Personal Health Records (PHRs) can be defined similarly to EHRs, except that patients can control them [11]. The aim is that PHR data enables the exchange of information between patients and healthcare professionals, through a standard used by different medical record systems and electronic devices that implement integration mechanisms between themselves. This standardization is primarily due to the provision of a database that aids medical analysis and diagnosis, enabling more accurate decision-making by professionals. The integration of PHR data, which includes patient-generated health data, is particularly valuable for personalized medicine, as it provides a comprehensive view of the patient’s health, lifestyle, and preferences, enabling truly individualized care.

Based on these points, it can be understood that integrating EHR data with PHR data within the same health system could provide patients and healthcare institutions with the resilience and ease of information exchange necessary to meet today’s data demands in this specific field of work. This integration is not only important for operational efficiency but also forms the foundation for advanced personalized medicine applications, enabling a comprehensive and dynamic understanding of each patient.

Having established this, we defined the following research question for this work:How can a health data management system be designed to seamlessly integrate EHR and PHR information while ensuring availability, reliability, and traceability?

This general research question can be subdivided into specific questions that will be addressed throughout the paper, such as which techniques to use for achieving the desired solution, how to design an architecture for this kind of system, how to store the clinical information required, how to develop mechanisms for tracing system’s events, which technologies apply to implement the system, and lastly, how to evaluate it.

To address these questions, this study proposes a decentralized architecture for developing electronic medical record platforms that integrate Electronic Health Record (EHR) and Personal Health Record (PHR) data. It incorporates a provenance model to enable access tracking and traceability. The proposed approach is designed to help healthcare organizations detect security breaches and prevent availability failures, ultimately reducing operational costs and minimizing harm to patients [12], while providing a cohesive and easily accessible digital system for medical facilities. By ensuring the integrity, availability, and traceability of patient data, this architecture facilitates personalized medicine, enabling clinicians to make data-driven decisions tailored to each individual.

Therefore, the primary objective of this work is to design an architecture capable of addressing common infrastructural problems in the eHealth sector, while also incorporating the necessary features to ensure data traceability and user activity auditing. With this, we intend to support the development of trustworthy eHealth systems that can meet the data requirements of personalized medicine. To support the solution, we developed an implementation based on the presented architecture and conducted a set of use case tests to verify its viability. The evaluations were designed to verify the system’s operation. To achieve this, we generated a dataset of synthetic clinical data for different patients over a specified time range and executed it through controlled data transmission using automation. The evaluation of the results aimed to understand which aspects of the developed system correlate with the desired solution and which do not, with a focus on qualitative observations rather than performance measures.

## 2. Related Work

In the work conducted in [13], a proposal is described for storing patient records in a distributed database, featuring the benefits of Medical Electronic Patient Records (EPRs) for clinical information management. It addresses medical records administration issues like availability and efficiency, which are solvable through distributed databases. The data is divided into Summary, Extended Summary, and Complete Medical Record levels for faster queries. Data distribution was controlled using GlassFish, and performance tests showed significant improvements in response time.

In [14], the AllHealthcare system is presented, which is a Personal Health Record (PHR) solution where the patient can build a Dynamic Monitoring Profile (DMP) for their healthcare. The aim is to give the patient control over their data so they can change and update their information in the health institution’s system anytime. The system was built as a mobile platform and uses the OpenEHR archetype standard to implement information models. The mobile application uses the PAD as an interface, which could be updated by generating a new template based on the OpenEHR standard. With the updated PAD, the new format is synchronized with the healthcare institution’s database, and thus new queries and user inputs could be supported. However, it should be considered that, despite using an edge application, the presented system defines a centralized database.

The health data sharing system proposed in [15] integrates blockchain technology and the decentralized file system IPFS. They highlight current security and infrastructure problems in health IT and present a blockchain-based solution with a customized consensus algorithm where network nodes store EHR and PHR data. The system, developed on the enterprise Ethereum blockchain Hyperledger Kiss as a security layer, uses IPFS for decentralization and storage. Despite not using standard data modeling norms, performance tests on transaction latency, scalability, and failure rate show the system’s efficiency. This work uniquely integrates EHR and PHR, emphasizing security through consensus mechanisms.

Considering the capture of provenance data, the work carried out in [16] presents a solution for securing health data using the OpenEHR standard, focusing on cloud computing and provenance techniques. The work proposes the specification of two complementary APIs to OpenEHR. The first is a Demographic API, focused on the management of patient demographic data, which is an aspect not addressed by OpenEHR. The second is a Provenance API that uses the audit and versioning data already recorded in OpenEHR to generate provenance documents compatible with the W3C PROV standard, promoting traceability and integrity of information. By integrating secure processing and provenance services in the health data management, this work contributes to advancing interoperability and security in OpenEHR-based healthcare systems. In parallel, our work objectives are to advance the features presented this work by taking into account a broader scope related to patient data and using provenance instrumentation techniques considering the context of data decentralization.

Each work analyzed uses concepts and technologies similar to those in the current work, but each one encompasses only a subset of them. As shown in Table 1, we focused on investigating works that were applied to the health sector and had been properly implemented. Work [13] diverges from ours in terms of infrastructure, as their approach utilizes distributed instances of the same database to address the challenge of creating a standardized system for EHR data. Work [14] provides in-depth details on the design and implementation of a dynamic edge application for storing health data that utilizes the standard and popular data modeling specification known as OpenEHR, only applied to the scope of PHR, with a centralized architecture. The work in [16] presents a comprehensive architecture that integrates standard OpenEHR health systems with custom provenance services, but also does not utilize a decentralized approach.

From all the works studied, the work in [15] was the closest to ours in terms of problem definition and solution hypothesis, presenting relevant results from the experiments. Our work aims to take a similar approach, but extend it to use provenance mechanisms to formal data traceability instead of a blockchain strategy. The use of standardized specifications to collect provenance data provides a structured and easy-to-query framework for historical information, including mechanisms that enable the detection of inconsistencies and failures in updates. The use of blockchain guarantees immutability and unauthorized access, but does not efficiently control end-to-end traceability. In this sense, the use of blockchain can be a storage solution for provenance data, but not a traceability solution.

## 3. Materials and Methods

To store *EHR* and *PHR* data in the same system, building a data model that would allow such integration is necessary. The template *International Patient Summary* [17], published on the *Clinical Knowledge Manager (CKM)* platform, was used as a reference to capture the information contained in a medical electronic record. *CKM* is an online collaborative platform developed to facilitate the management of clinical artifacts. The artifacts include clinical data models implemented in health information systems, especially those based on *OpenEHR*. In addition to this template for *EHR* data, another template was used to represent PHR data, based on the ongoing work presented in [18].

### 3.1. Data Design

**Figure 1:**
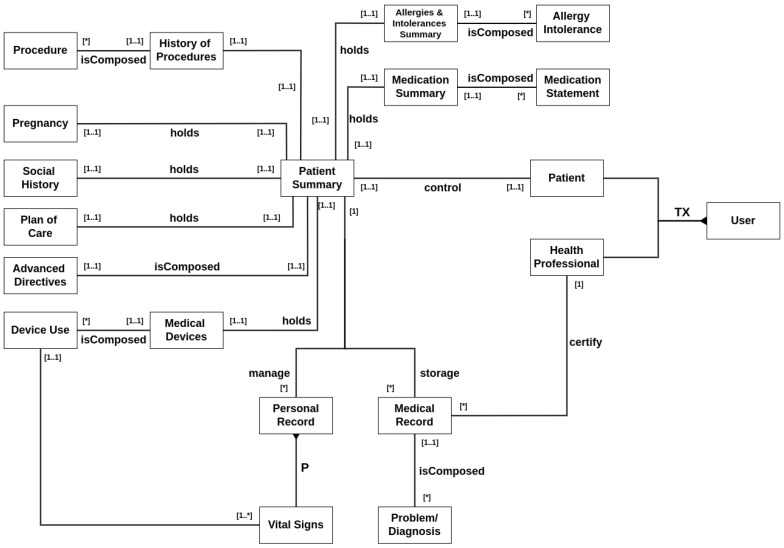
Entity–relationship diagram of data. The asterisk symbols (*) in the diagram relationship denotes an arbitrary number of one entity in relation to another (e.g., A single History of Procedures entity can be composed of any number of Procedure entities).

Figure 1 illustrates a conceptual diagram that represents the information. It was structured as follows: Aside from administrative accounts, two types of users can be registered in the system, patients and health professionals. A patient controls exactly one summary, which is his own. A patient summary manages three types of information: patient details, medical records, and personal records.

Patient details include several types of information. The history of procedures manages past operations, surgeries, emergencies, etc. History of allergies is for registering current or past patient allergies. A medication summary describes the prescriptions made for the patient. It also covers pregnancy records, social history reports about the patient’s habits such as smoking and drinking, resumed information about its plan of care, advanced directives for anticipated care engagement, and finally, a list of any possible device uses the patient has.

Medical records are general documents created during examinations, appointments, clinical meetings, or any type of interaction with a healthcare professional or institution. A healthcare professional must certify a medical record when it is created, and a patient can have only one collection of medical records.

Finally, personal records are any data a patient wants to share with a healthcare professional. The primary type of personal record is the patient’s vital signs, which encompass various physiological measurements, including temperature, blood pressure, and oxygen saturation, among others. A patient can have multiple collections of personal records regarding different types of information.

This data design was built around a decentralized system that utilizes two distinct data repositories: one designated for stable information, such as medical records, and another for dynamic, high-frequency personal data. This configuration enhances query performance while addressing challenges like pinpointing the appropriate data source and translating abstract user requests into precise query formats.

The design adopts a unified schema supported by a logical integration strategy. User interactions occur through a mediation layer, which is responsible for managing queries, locating relevant sources, dispatching requests, and formatting the returned data. This intermediary layer interfaces with the data repositories through adapters that reformat queries into source-specific structures and convert responses as needed. Additionally, the architecture aims to store healthcare information in a decentralized manner, utilizing distributed technologies to distribute new data entries across multiple network nodes, thereby enhancing data accessibility and protection.

### 3.2. Architecture

**Figure 2:**
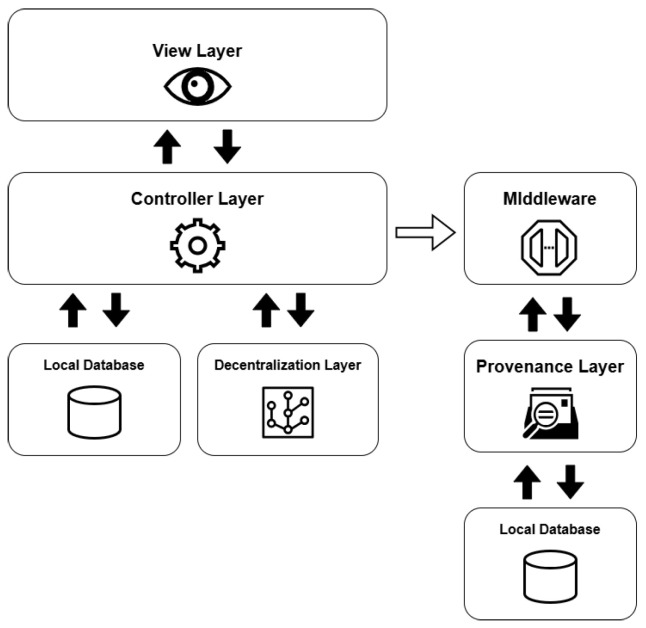
Simplified diagram of the architecture and its layers.

The necessary components and responsibilities were organized into different layers to design the architecture, as shown in Figure 2.

The view layer serves as the access point through which users interact with the system’s information, offering visual interfaces to submit queries and view their corresponding results. Implemented as a *REST API*, this layer facilitates communication between the user and the platform via HTTP requests and responses, primarily using the *JSON* format for data exchange. In addition to these user-facing endpoints, the system also includes a management interface designed for administrators to supervise and control data operations.

The controller layer is responsible for overseeing and verifying the data maintained within the repositories. It also incorporates *Data Access Object (DAO)* patterns to handle the manipulation of the entities defined in these data stores. Additionally, this layer handles tasks related to user verification and access control. This layer is also responsible to send the events notifications to the middleware so it is able to register the related provenance information.

The decentralization layer handles both the coordination of database integration and the execution of decentralized operations. It oversees the entire life cycle of the data sources and implements the corresponding *wrappers* for each. Designed as a form of *middleware*, this layer enables the flexible attachment and detachment of various types of data repositories. Within this structure, the medical data repository was developed using *IPFS* technology, while the personal information repository was implemented with the *GUNJS framework*.

The provenance layer is tasked with collecting, recording, and querying provenance-related information about every event that takes place within the system. To achieve this, it was developed as an instrumentation service (an *API*) that monitors system activities and stores related data following the *PROV* standard.

**Figure 3:**
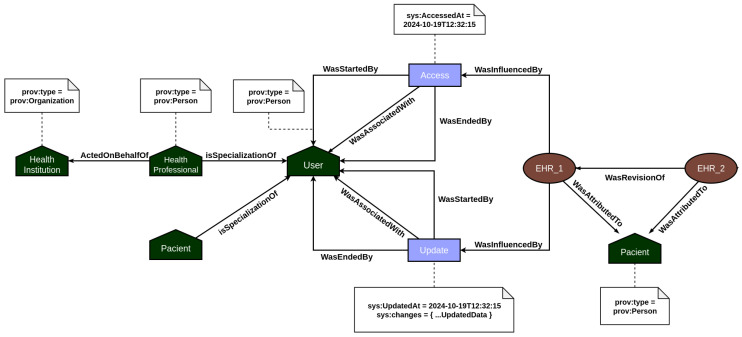
PROV model mapping the EHR’s relationships between documents.

**Figure 4:**
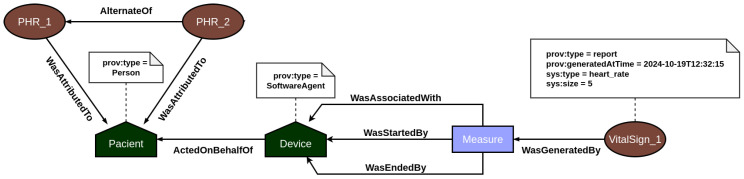
PROV model mapping the PHR’s relationships between documents.

The provenance layer main objective is to offer a reliable mechanism for tracking and auditing the data handled by the platform, particularly focusing on *EHR* and *PHR* information. The representation of relationships among agents, activities, and entities—core elements of the PROV model—is illustrated in Figure 3 and Figure 4. Built using the Strapi framework, this layer saves PROV records and their associations in a local data repository. It captures every incoming system request and applies all predefined causal relationships structured within the application model.

In EHR data provenance, the primary actors are the patient and the healthcare professional. EHR data is attributed to the patient, but the healthcare professional acting on behalf of a healthcare institution is responsible for updating the patient’s medical record information. In addition, both the act of accessing and updating the medical record by the users are recorded in the provenance, so that it is possible to consult individuals who analyzed and changed the data and investigate what changes were made. On the other hand, PHR data provenance focuses on employing the traceability of vital sign data generated by sensors. In this context, the primary actors are the patient and their vital sign collection devices. Every time a measurement event is recorded in the system, the provenance service is responsible for recording all documents associated with the measurement, such as the device used, and the start and end of the measurement, among other contextual data. In this way, the user’s PHR data is incremented over time with the information on the vital signs collected.

The database receives all queries made in the system in a mirrored manner and performs updates to the data locally. This way, if the system fails at any time, or it is necessary to perform an audit on the information transferred between the network nodes, the local database will serve as a backup.

In order to synchronize the eHealth system with the provenance system in a modular way and with a low level of overlap between the layers, a middleware layer was defined, whose responsibility is to receive from the controller layer the events that have occurred with the data and generate the appropriate provenance documents that map and record them. In this way, this middleware layer has the function of creating the provenance instrumentation designed for the architecture, assembling a new document from the notified event, and registering it in the provenance layer.

The controller layer forwards to the middleware layer events associated with requests that users have made from the visualization layer, which can be requests to create, update or access data.

The intermediate layer plays an essential role in the modularity and organization of the architecture, since it contributes to the decoupling of the eHealth and provenance systems, thus keeping the main rules of operation between them in a single module that can be adjusted independently.

### 3.3. Infrastructure

To construct the system, several modules were developed to handle the HTTP operations in a RESTful API, manage user authentication, and control database entities. The services were created using https://strapi.io/ (accessed on 9 June 2025), as mentioned before, which is an open-source content management system (CMS) that empowers developers to efficiently generate, organize, and distribute content within API-centric solutions. Strapi’s adaptable and configurable nature supports the development of personalized REST APIs, allowing for the definition of data models, relationships, and validation layers.

Strapi speeds up the setup of data models and their associations by automatically generating views, HTTP endpoints, and data handlers. To enable the decentralization layer, custom logic was introduced via middleware, allowing distributed query processes to run on routes linked to medical and personal record data. Dedicated middleware components were created for both OrbitDB and GUNJS—where OrbitDB manages medical record storage, and GUNJS handles personal data storage. OrbitDB operates as a decentralized database tailored for peer-to-peer environments utilizing IPFS; it supports multiple types of databases and guarantees data authenticity and protection through cryptographic methods. Meanwhile, GUNJS is a real-time, decentralized database toolkit that synchronizes data across devices without relying on a central server, employing a graph-based structure and offering encryption support.

**Figure 5:**
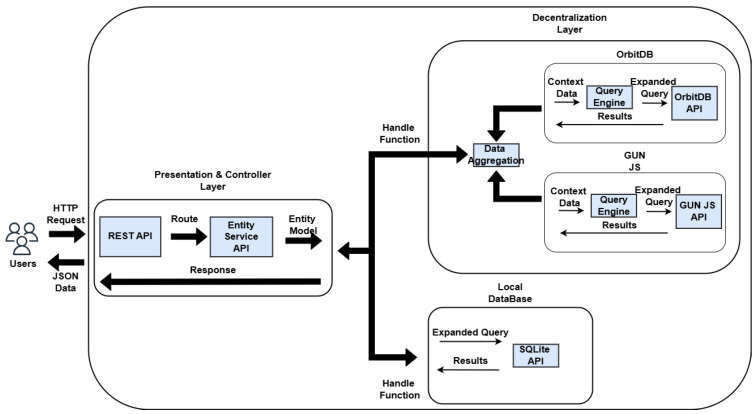
Infrastructure built for the system and its flows.

The core modules communicate as follows: the user sends HTTP requests to the RESTful API server managed by Strapi, which subsequently triggers the appropriate route and handler. These handlers then leverage the Entity Service API to carry out actions on the underlying data stored in the database (Figure 5).

When a request is made for either a medical or personal record, a process activates the middleware to replicate the query across decentralized data repositories. This middleware module accepts an object containing all relevant contextual details—such as route, parameters, filters, and access credentials—which are then utilized by a query processor to generate tailored queries for each specific database. The outcomes of these queries, retrieved from either local or distributed sources, are delivered as a JSON-formatted response.

**Figure 6:**
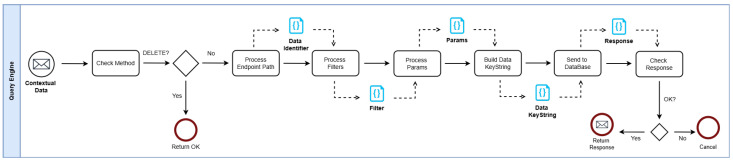
The proposed architecture with its major components and workflow.

Due to the tools used to develop the decentralized system, there is no guarantee the data can be removed from all nodes in the network, as there could be situations where a node is disconnected when the delete operation is executed, for example. Therefore, the policy adopted was not to implement the delete operation for the decentralized databases. This behavior was mainly built into the query engine, as can be seen in Figure 6.

Initially, the system verifies whether the incoming request uses the DELETE method. If so, the engine halts the transformation process and terminates the operation. Subsequently, the request’s identifiers, filters, and parameters are combined to generate a keystring that uniquely identifies the target element of the query. This keystring is then forwarded to the corresponding data repository. In the event of a successful operation, the result is passed back to the middleware layer. If the process fails, the query is aborted to prevent any disruption to local processes. Additionally, every user request is logged in a local storage system to guarantee data durability.

For GET operations, the system initially checks the local data repository. If the desired entry is not located, the search proceeds within the decentralized storage. A specific parameter may determine whether the lookup should remain local or extend beyond. In the case of POST operations, data is added to the decentralized store only if the insertion into the local database succeeds, ensuring valid input and establishing basic synchronization across both sources. PUT operations follow a comparable strategy to GET: if the item targeted for modification is not found locally, the system searches the decentralized repository and performs the update if the item exists. These mechanisms collectively support the maintenance of data integrity and alignment between the local and distributed databases.

The provenance layer was also built using the Strapi platform. Since we defined the PROV model as the provenance registration method, the API’s data modeling was based on it. PROV documents were designed with the same specifications as PROV’s agent, entity, and activity objects. In addition, the main causal relationships were constructed as objects that relate to the above mentioned objects so that they can also be consulted. The API uses the REST format for its requests. Therefore, any objects and their relationships are managed through HTTP requests of the types GET, POST, PUT, and DELETE.

The middleware layer was developed as a *REST API* that opens an HTTP route for the eHealth system, enabling it to send event notifications via webhook. The middleware was build using the *Express JS* app framework, and it also connects to the provenance service to send requests, primarily registering the PROV documents generated from the events.

To provide authorization mechanisms for the system, we developed administration accounts to manage the underlying routes and interaction permissions for users. For a patient or health professional to use the application, they first need to register their credentials. These types of data are stored with encryption to ensure that no one but the user knows the information. Once this is complete, the user will need to log in to the system, which essentially involves making a request with their email and password. As a response, they will receive an access token. This token can then be used in the following system’s interactions, attaching it to the request.

The system administrator is capable of assigning roles to users. Additionally, they are capable of configuring each role to access predefined sets of routes, and also specify which type of request they can make in that route.

### 3.4. Evaluation

In order to verify if the system meets the required functionalities designed, we established that it was necessary to evaluate how the solution would behave when receiving requests containing real data. However, having access to large samples of clinical data from real patients is difficult due to its sensitive and private information. So to overcome this problem, we decided that for the current state of the work, synthetic clinical data would be adequate, and to generate the required data we used a tool called *Synthea*. *Synthea* is an open-source software tool that generates high-quality, clinically realistic synthetic patient health records [19]. It simulates the entire lifespan of synthetic patients, modeling various aspects of healthcare, including medical conditions, treatments, and outcomes, based on publicly available data and clinical guidelines. By producing synthetic data that mirrors real-world health records without compromising patient privacy, Synthea enables researchers and developers to conduct scientific experiments, validate eHealth applications, and train predictive models without the legal and ethical constraints associated with real patient data. The generated data can be exported in multiple standardized formats, such as *HL7 FHIR*, *C-CDA*, and *CSV*, facilitating interoperability and integration into various health IT systems.

**Figure 7:**
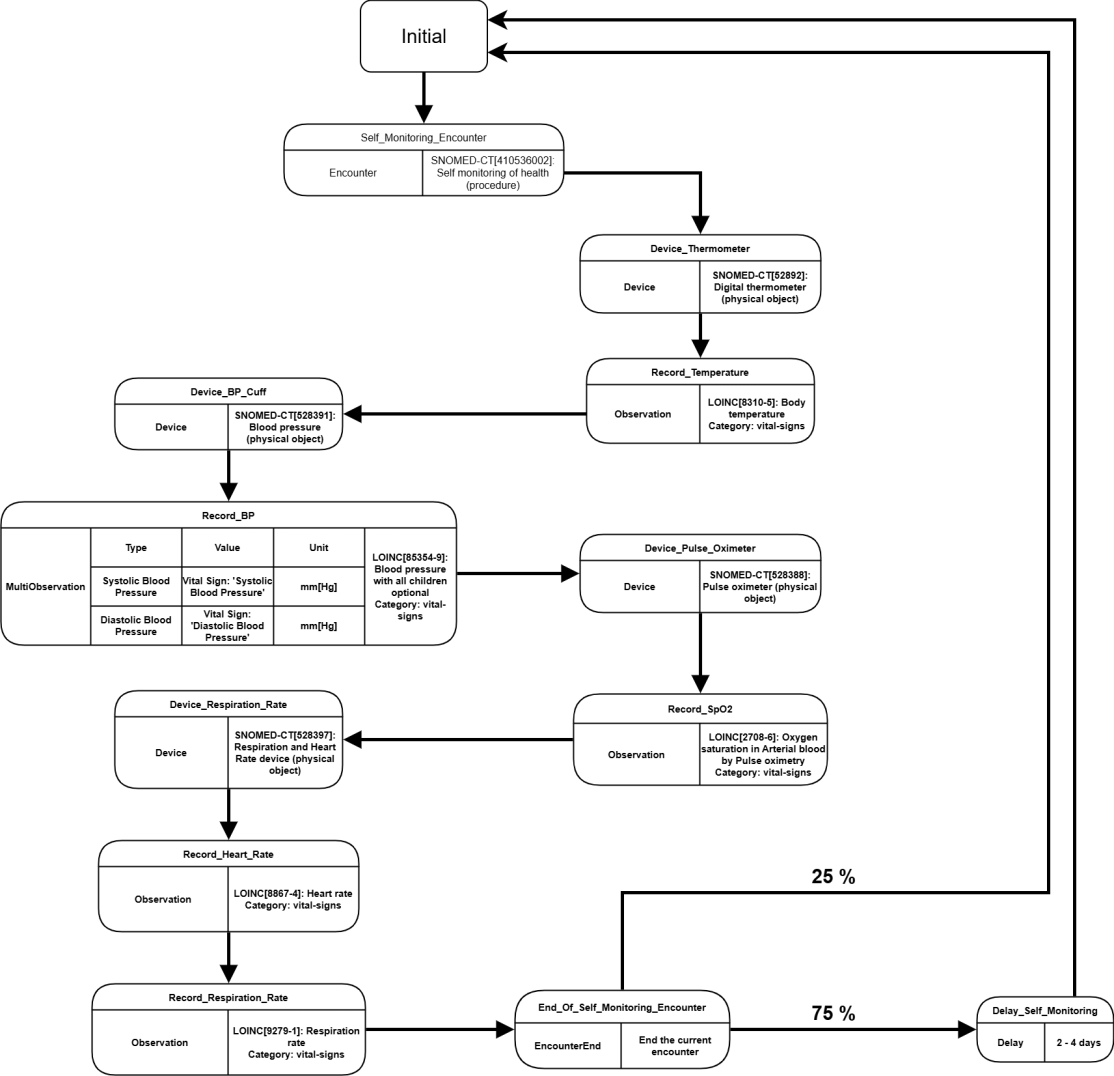
Flow Diagram presenting *“Self Monitoring Wellness”* custom module.

The default modules present on Synthea do not have the capabilities to generate data where patients perform self measures of their vital signs, which would be a setback for our *PHR* test. So, to fabricate a scenario where the patients have this behavior, we developed a custom module for this experiment called *“Self Monitoring Wellness”*. This module cycles along during the patient’s lifespan and simulates an encounter where it is assumed the patient takes several measures of their vital signs, using the associated measuring devices, as in Figure 7.

During the patient’s lifespan, the custom module cycles over the *Self Monitoring Encounter* and creates records of device use to measure different types of vital signs, such as temperature, heart rate, oxygenation, etc. Each vital sign’s record is associated with the encounter and the device, describing the measure’s code, type, and value. After ending the encounter, there is a 25% probability that another encounter will happen on the same day, taking a new set of vital signs in a different time period. Otherwise, the encounter generation cycle is delayed for a period between 2 to 4 days. The module was defined this way exclusively to avoid a large volume of vital signs, which could harm the system’s performance in general. The Synthea was executed using the parameters shown in Table 2.

With the synthetic data generated, the next step was developing an automation that could read the datasets and perform requests for the system. To achieve that, a Python [20] script was developed, focusing primarily on registering in the eHealth system the data related to organizations, health professionals, patients, devices, medical records, and personal records. The script also ensures that each instant is related to each other by references, which is essential for the provenance service to work correctly. In addition, due to limited computational resources, only a sample corresponding to one month of the original dataset generated was used during the tests, specifically in relation to the vital signs entries.

## 4. Results

### 4.1. Health Dataset

**Figure 8:**
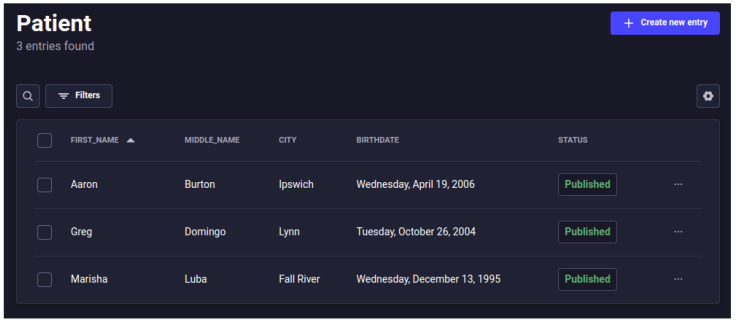
Screen from the Strapi Content Manager, showing the registered patients.

As seen in Table 3, 190 instances of the central entities were registered in the eHealth system. Three patients were registered as specified in the Synthea configuration, also shown in Figure 8, and about 28 other entities, representing the health institutions and their members, were recorded. The 12 devices created correspond to 4 devices (thermometer, blood pressure, oximeter, and heart rate measurer) for each patient. The 123 vital signs registered correspond to 36 records from the patient *Aaron*, 42 records from the patient *Greg*, and 45 records from the patient *Marisha*.

Table 4 shows the amount for each of the main information entities registered in the provenance service. A total of 573 entries were registered, including other PROV documents. Each activity and generation corresponds to a record of a vital sign, and each entity corresponds to the creation of it, in addition to the entities related to *EHR* and *PHR* documents. The associations are between the measured activity and the device that performed the measure, as explained in Figure 4.

### 4.2. Health Professional System Access

Another feature of the system developed is to record the provenance of data access by health professionals. To test whether this mechanism was designed as expected, one of the users registered as a healthcare professional was selected to serve as an actor to show a request for information from a specific user. For this scenario, the healthcare professional registered as *Agnes Dooley* was used, granting her the appropriate permissions to access patient data in the eHealth system. She then submits a request to access the information of patient *Aaaron Burton*.

**Figure 9:**
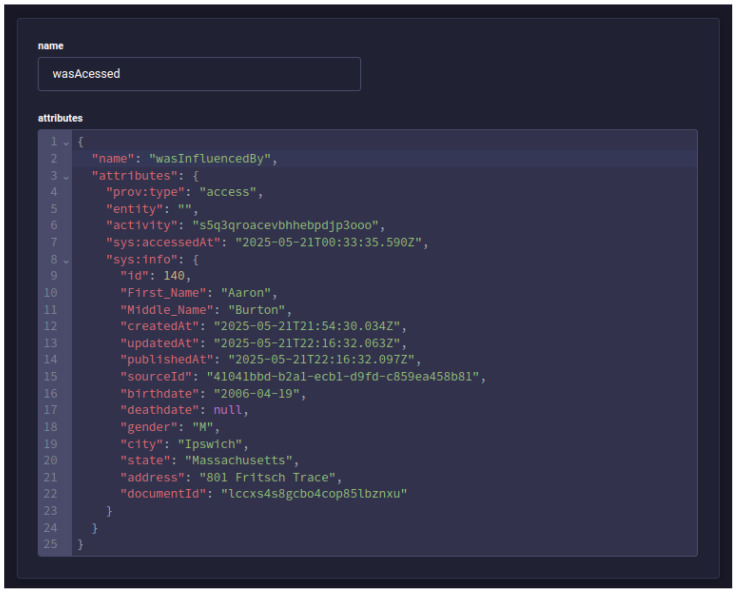
Screen from the Strapi Content Manager, showing the PROV document registered for the access performed by the health professional.

After making the data request, five new PROV documents were generated in the provenance service as expected: a *sys:ehr-access* activity, an access start entity, an access end entity, an association of the activity with the agent that executed it, and finally an influence of type *wasAccessed* with the information of the user who made the access and what was accessed, as shown in Figure 9.

This example shows that recording events occurring in the eHealth system using the provenance instrumentation generates a high level of data flow traceability in the system. Specialized queries in the provenance service could easily identify the source from which a piece of data was generated, the context in which it was created, the participating users, and many other relevant information for possible investigations and audits. Therefore, considering this evaluation, we could answer the research question previously defined. This was possible considering that we use specific technologies, such as a decentralized data storage architecture and provenance models to support our approach.

## 5. Discussion

This study presents a decentralized solution for unifying *EHR* and *PHR* repositories within healthcare organizations. The proposed architecture and its layers were thoroughly described, along with the developed system and the technologies employed. In conclusion, functionality evaluations were conducted to validate the application’s performance, and a provenance scenario was analyzed. This article also extends previous works that aim to evolve the approach to the problems exposed and refine the proposed solution [21].

The solution presented has some advantages over the most common current platforms. Firstly, the solution has the ability to continue operating even after experiencing losses in multiple network nodes. This approach brings a high level of availability and resilience to the area of healthcare services that is still little addressed by existing products, such that the new proposal offers a better chance of remaining operational under external attacks from malicious entities or infrastructure failures. This continuous availability ensures real-time data access and comprehensive patient information for timely and tailored interventions, thereby avoiding disruptions in care that could impact individual treatment plans from a personalized medicine perspective.

In addition, the data provenance recording features in the architecture add significant value to the reliability of systems built on top of it, considering that this technology enables the tracing of the origins and history of data that passes through the system. The PROV model provides a standardized approach for recording and analyzing the life cycle of a digital artifact and its actors, enabling an examination of how it reached its current state. This type of understanding is especially valuable in the healthcare sector, as both patients want to understand how certain documents and diagnoses were made, and healthcare professionals need to know the origin of the information attributed to their patients.

Another positive aspect of the proposed solution is that it is primarily modeled as a *headless* system, i.e., it does not have an end-user operating interface in the foreground. It was modeled in this way mainly on the premise that other IT systems would already be in operation in the respective target healthcare institutions, and the proposed solution could then be easily integrated with them via intermediary services. This guarantees both a low level of coupling and installation costs, as well as high ease of maintenance between the systems in place. This interoperability enables the seamless integration of diverse data sources (e.g., electronic health records, wearable device data, genomic sequences) from various existing systems into a unified and trustworthy platform for personalized medicine, creating comprehensive patient profiles.

Although the evaluation presented demonstrated the feasibility of the architecture, some essential functions could not be properly assessed due to limited computing resources. The system was executed on a local machine, which had a 2-core CPU at 1.90 GHz and 11.5 GB of RAM. Therefore, performance tests associated with data decentralization have not yet been possible, and this is a task for future work.

One possible issue observed in the conducted executions of the system is the high ratio between the generation of clinical data and the generation of provenance data. Based on the number of entries presented in Table 3 and Table 4, the total ratio between the two types of data is approximately 3.1211. However, the volume of provenance data could be even greater in larger systems because it is generated not only when data is created, but also when it is accessed or updated. Better approaches for data storage and compression for the provenance service are currently being investigated.

This article employed synthetic data to encompass a broad range of patient types and health conditions. This extensive dataset was crucial for verifying the viability of the proposed solution across diverse scenarios and ensuring a robust assessment of its capabilities. We recognize the importance of validation with real data, and this will be our next validation cycle. However, at this first cycle, it was essential to verify the viability of the architecture to capture provenance, considering all types of data and structures. This would not be possible in a real environment, where data is generally more specific and typically lacks outliers that help validate the most challenging cases.

For this reason, performance metrics were not prioritized in the architecture feasibility study, as our goal was to verify the viability of the solution in addressing the proposed problems. However, studies are already being conducted, using the *REPESQ* computational infrastructure [22] to address reliability and scalability metrics, among other important non-functional requirements.

Additionally, one relevant drawback for the current work is the lack of a group of volunteers who could provide real data to validate the solution. This would improve the experiment both technically and analytically, as we could measure performance associated with the users’ requests and the volume of data generated in a real environment, and better understand how patients and health professionals interact with the system, thereby collecting valuable feedback. Real-world data and user feedback will refine the system to meet other requirements of personalized medicine, including patient engagement and clinician workflow optimization. In the next validation cycle, we are selecting participants for feature validation, as well as proceeding to assess the research with the ethics council for the proper experimentation.

Other implementation-related challenges remain to be explored in depth. One aspect is evaluating how interoperability between current healthcare systems and the proposed solution can be achieved to enable a smooth and secure transition. Additionally, the system must be analyzed from a legal perspective to ensure compliance with relevant regulations and standards, particularly those concerning patient data privacy and protection.

It is important to observe that the synthetic data generated by Synthea consists of anonymized data and fully complies with Brazil’s General Personal Data Protection Law (*LGPD*). According to Article 12 of the *LGPD*, anonymized data are not considered personal data, except when the anonymization process is reversible using proprietary means or “reasonable efforts” [23]. The law further clarifies that what constitutes “reasonable efforts” must be evaluated based on objective factors, such as the costs, time, and technologies available.

In the next evaluation cycle, when we begin working with real-world data, we will comply with the *LGPD* by obtaining all necessary legal authorizations, including informed consent from patients or other applicable legal bases, as required by law. We are currently in the process of securing these permissions. However, even in this second phase, all data will undergo irreversible anonymization using up-to-date technical measures (e.g., suppression, generalization, masking, or noise injection), ensuring that re-identification, whether direct or indirect, is not feasible, even with reasonable efforts.

Thus, throughout both phases—first with synthetic data and later with real data—we ensure full compliance with the *LGPD* regarding the anonymization of data. Patient identities cannot be recovered or correlated under any circumstances.

Several achievements are going to be pursued in the future. One of the goals is to deploy the system in an environment with a larger number of interconnected nodes—both within the same network and across different ones, to assess scalability, availability, and performance. Another issue involves potential synchronization inconsistencies between local and distributed data stores under unstable network conditions. The objective is to subject the system to these scenarios, monitor its response, and introduce enhancements if any issues are identified, ultimately leading to more resilient platform versions. Furthermore, we plan to extend the services of the provenance layer by enhancing the granularity of captured data and applying machine learning techniques to derive insights from provenance records. Leveraging machine learning on rich provenance data can make new possibilities available for personalized medicine, allowing for more adaptive and predictive models based on the complete history and context of patient data. Lastly, integrating a decentralized solution is planned to securely store provenance information.

## 6. Conclusions

There have been numerous efforts in the recent literature to find solutions that improve infrastructure resilience, data interoperability, and functional user experience in healthcare systems. This demonstrates that the current problems in the scope of eHealth and personalized medicine are rising concerns for several institutions. In this work, we presented an architecture for *EHR* and *PHR* data management that addresses these issues by utilizing decentralized technologies and provenance mechanisms. This approach enables the building of trustworthy and comprehensive patient data foundations necessary for truly personalized healthcare. Although the experiments conducted could not verify the performance and efficiency aspects of the proposal in detail, the results obtained from implementing this architecture can support the hypothesis that promoting this type of system design is a viable solution for the defined problems, ultimately paving the way for more precise and individualized patient care under the personalized medicine perspective.

## Figures and Tables

**Table 1:** Comparison table highlighting the differences between the related work analyzed and this article’s solution.

Work/Proposal	EHR	PHR	Provenance	Decentralization	eHealth	Implementation
[13]	X			X	X	X
[14]		X			X	X
[15]	X	X		X	X	X
[16]			X		X	X
Our Work	X	X	X	X	X	X

**Table 2:** The description of each parameter used to generate the synthetic data on Synthea.

Parameter	Value
Population	3
Age	[18, 30]
Reference date	1 May 2025
End date	1 June 2025
Seed	123
Clinician seed	123
Append numbers to person names	False
Years of history	1
Exporter.csv.export	True

**Table 3:** Table of entries registered in the eHealth service.

Instance	Number of Entries
Organizations	14
Health Professionals	14
Patients	3
Devices	12
Medical Records	24
Vital Signs	123

**Table 4:** Table of entries registered in the provenance system.

Instance	Number of Entries
Activities	123
Agents	43
Entities	129
Delegations	26
Associations	41
Generations	123

## Data Availability

The data presented in this study are available on request from the corresponding author.

## Abbreviations

The following abbreviations are used in this manuscript:

API	Application Programming Interface
CMS	Content Management System
EHRs	Electronic Health Records
HTTP	Hypertext Transfer Protocol
PHRs	Personal Health Records
P2P	Peer-to-Peer
IPFS	Interplanetary File System
